# Titanium matrix composites reinforced with biogenic filler

**DOI:** 10.1038/s41598-022-12855-5

**Published:** 2022-05-24

**Authors:** Izabela Zglobicka, Rafal Zybala, Kamil Kaszyca, Rafal Molak, Monika Wieczorek, Katarzyna Recko, Barbara Fiedoruk, Krzysztof J. Kurzydlowski

**Affiliations:** 1 Faculty of Mechanical Engineering, Bialystok University of Technology, Wiejska 45C, 15-351 Bialystok, Poland; 2 Lukasiewicz Research Network—Institute of Microelectronics and Photonics, Al. Lotnikow 32/46, Warsaw, Poland; 3 Faculty of Materials Science and Engineering, Warsaw University of Technology, Woloska 141, 02-507 Warsaw, Poland; 4 Faculty of Physics, University of Bialystok, K. Ciolkowskiego 1L, 15-245 Bialystok, Poland

**Keywords:** Mechanical engineering, Structural materials, Ceramics, Composites, Mechanical properties, Metals and alloys, Characterization and analytical techniques, Design, synthesis and processing, Techniques and instrumentation, Imaging techniques

## Abstract

Novel metal matrix composites (MMCs) have been fabricated with Ti6Al4V matrix and a biogenic ceramic filler in the form of diatomaceous earth (DE). Mixtures of DE and Ti6Al4V powders were consolidated by the spark plasma sintering (SPS) method. Microstructure of the consolidated samples has been investigated with microscopic techniques and XRD. Thermomechanical characteristics have been obtained using small-sample techniques. The results obtained indicate that the fabricated composites show outstanding mechanical and thermal properties due to synergic effects between the filler and the matrix (beyond the rule of mixtures).

## Introduction

Metal matrix composites (MMCs) are a new class of engineering materials of tunable mechanical and functional properties^1^. One of the most frequently used matrices of MMCs is titanium and titanium alloys, such as dual-phase Ti6Al4V^2^.

Widely used reinforcements of Ti alloys-based composites reported in the literature are: TiB, TiC, TiB_2_, TiN, B_4_C, ZrC, SiC, Al_2_O_3_, and carbon nanotubes^3–7^. Because of the high chemical reactivity of Ti during the conventional ingot metallurgy process, but also to reduce the cost and material loss in the manufacturing process, the commonly employed method of manufacturing TMC with discontinuous filler (particles or short fibers) is powder metallurgy (PM)^8–10^. The key parameters that ensure good composite performance are homogeneous dispersion of reinforcement and high adhesion to the matrix.

Depending on the reinforcement and matrix reactions, ex-situ and in-situ fabrication methods may be distinguished^11^. Composites with thermodynamically stable ceramics, such as SiC, TiC, TiB, or ZrC, are processed ex-situ. This route does not change either the particle size or their morphology and results in superior mechanical properties (wear resistance and friction coefficient under dry sliding conditions, etc.). The reactivity of the titanium matrix with boron, carbon and nitrogen allows for in-situ processing. The better interfacial bonding obtained by in-situ methods results in enhanced tribological performance of these composites.

Furthermore, there are two possible approaches of MMC in PM, known as the blended elemental (BE) method and the pre-alloyed (PA) powder method^8,12^. The elements obtained via the BE method show lower mechanical properties, whereas the mechanical properties of MMCs in PM manufactured by the PA method are comparable to those produced with Ti alloys^8^.

Wrought Ti6Al4V alloy exhibits tensile strength in the range of 850–1200 MPa, with ductility between 3 and 26%^8,13–16^. Tensile strength of PM Ti6Al4V depends on the porosity and microstructure.

Element sintered by BE imparts strength in the range 750 to 900 MPa^8,17–20^ with elongation 3 to 13%^8,17–20^. PA Ti6Al4V exhibits a wide range of the tensile properties – 700 to 1070 MPa with 7.5–21% for ductility^8,17,21–24^. The higher limit of strength is obtained for PA elements with 100% density^25^.

Ti is also known to react with Si, and because of the beneficial effect of Si addition on the oxidation and creep resistance of Ti-X-Si alloys, Ti-Si systems continue to attract technological interest^26,27^. The equilibrium phase diagram indicates five silicide phases, four fully stoichiometric (TiSi_2_, TiSi, Ti_5_Si_4_, and Ti_3_Si), and one non-stoichiometric (Ti_5_Si_3_). Metal silicides, among intermetallic compounds, are generally considered as imparting good mechanical/physical properties^28^.

The potential source of Si may be silica (SiO_2_) which occurs in different types, i.e., fumed silica, precipitated silica from alkali silicates, clays, glass as well as silica from the dissolution of minerals^29–36^.

As previously mentioned, Si-rich fillers for advanced composite materials are diatom frustules. There has been a systematic growth in the number of publications reporting advanced applications of diatoms in recent years^37^. The interest in material properties of diatoms is determined by their unique hierarchical organization with micro- and nano-sized open volume. Geological deposits of diatoms are called diatomaceous earth. DE deposits are mined from in many places in North America, but exist on every continent except Antarctica.

Because of the unique hierarchical architecture of diatom frustules, it is desirable to search for the technological route which preserves their morphology after being incorporated into a Ti-matrix. Spark Plasma Sintering (SPS) is the new method of fabricating TMCs, which allows fabrication of fully dense composite under high heating rates, relatively low average temperatures, and short processing time. Furthermore, it allows combines the effects of mechanical loading, temperature and electric current, which all together results in effective bonding between particles and the matrix. The diameter of the preforms which can be used for fabrication in SPS is up to 300 mm^38–40^.

Available data indicate that SPS consolidation of Ti6Al4V has been performed at temperature range of 700–1500 °C and compaction pressures from pressure-less to 80 MPa. A heating rate of 100 °C/min and a holding time ranging from 2.5 to 20 min was applied^41–45^.

In our approach, the effect of silica diatomaceous earth reinforcement on the microstructure, mechanical as well as thermal properties of composites manufactured by Spark Plasma Sintering (SPS) is presented. To our best knowledge, so far, there have been no literature reports about the manufacturing of MMCs with Ti matrix with diatoms frustules as an additive.

## Results and discussion

### Powders characterization

**Figure 1:**
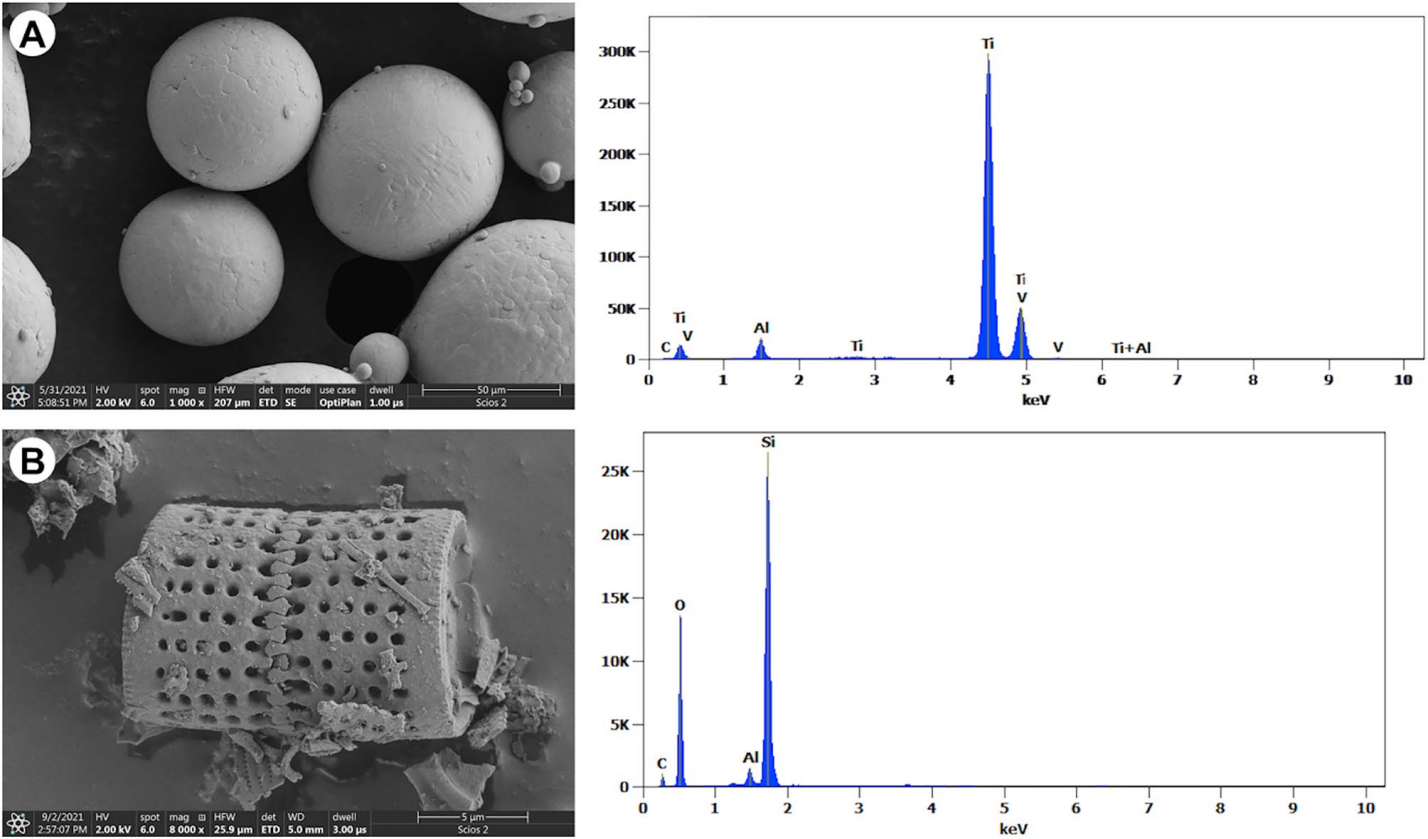
SEM–EDS morphologies of as-received (**a**) Ti6Al4V particles and (**b**) diatomaceous earth (DE).

SEM images of as-received Ti6Al4V and diatomaceous earth material are presented in Fig. 1.

Ti6Al4V particles (Fig. 1A) are spherical and non-porous, with some satellites attached to the larger ones. The EDS spectrum confirms that the titanium powder consists of Ti, Al, and V (Fig. 1A).

A single shell from diatomaceous earth is characterized by a regular, cylindrical shape with small holes on the cylinder walls (Fig. 1B). It is of the genus *Aulacoseira*, a common representative in DE of freshwater origins. The EDS analysis of the diatomaceous earth in Fig. 1B confirms the presence of Si and O (the occurrence of Al is an artefact).

Laser particle size analysis determined the average diameter of Ti6Al4V: 86.23 ± 0.19 µm, which agrees with the manufacturer’s data. Within the diatomaceous earth, particles in the range 4.47–517.20 µm can be distinguished. The mean size of filler particles is 26.32 µm.

### Composites

#### Relative density

The relative density of all spark plasma sintered samples was 100%, disregarding the content of DE. Such high relative density of the material imparts good mechanical properties and performance. The exact values of the theoretical as well as experimental density have been provided in the Supplementary Information (see Table S1). When analyzed with the principle of the rule of mixing, for all composite samples the measured densities are higher than theoretical values. On the other hand, for pure Ti-alloy experimental value is slightly lower (relative difference of 0.71%). The latter indicates some residual porosity, which is below the resolution limit of SEM observations performed. The higher than theoretical values of density for composite samples might be related to changes in the structure/morphology of DE under the consolidation conditions.

#### Scanning electron microscopy observations

**Figure 2:**
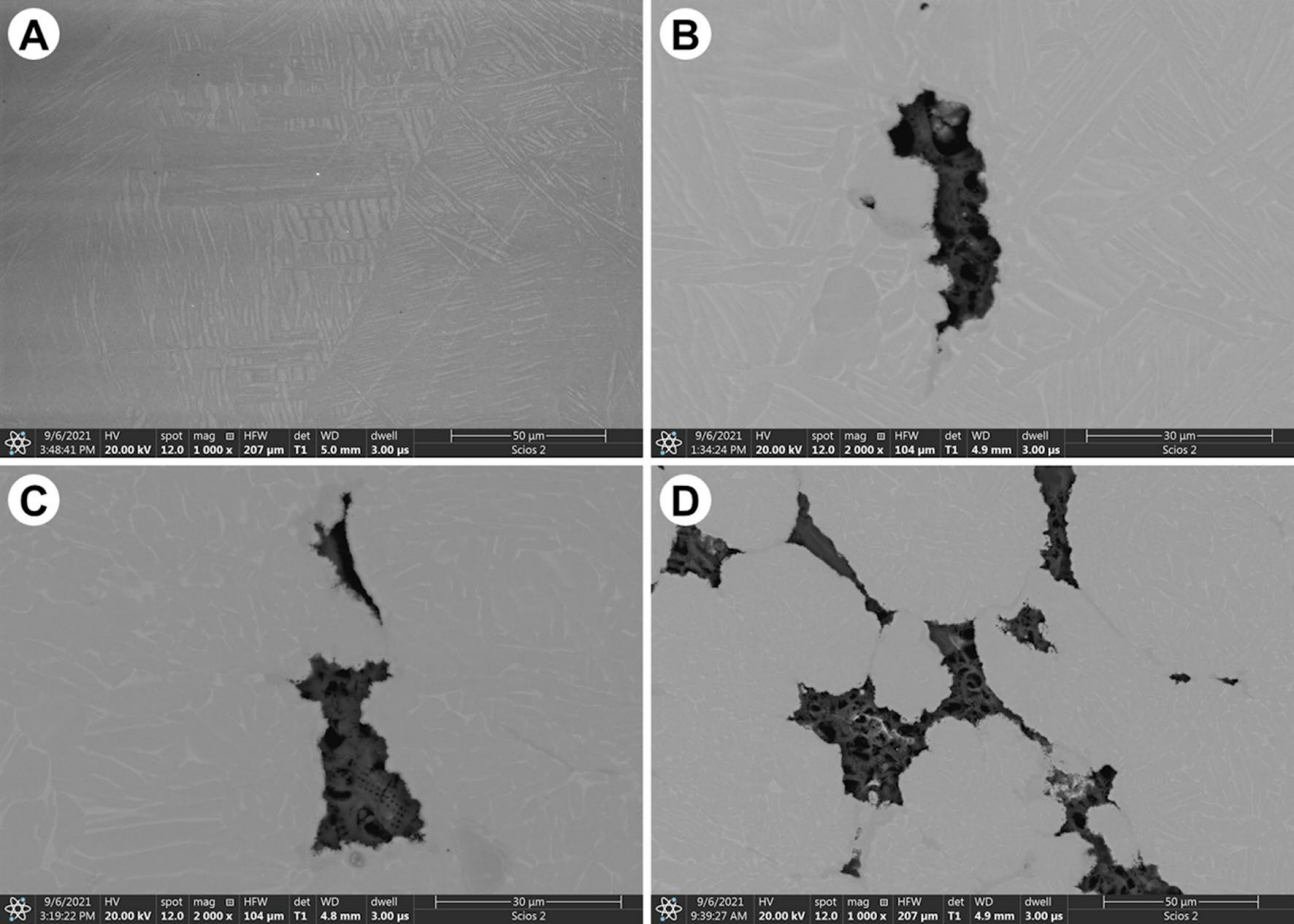
SEM images in the BSE mode of composite: (**a**) Ti6Al4V alloy, (**b**) Ti6Al4V/1% DE, (**c**) Ti6Al4V/5% DE, (**d**) Ti6Al4V/10% DE.

SEM images of diatomaceous earth-reinforced Ti6Al4V alloys are shown in Fig. 2. The backscatter mode reveals in the metallic matrix two phase lamellar structure, which is characteristic for the Ti6Al4V alloy^7,46^. The SEM image of the sample without addition of DE (Fig. 2A) proves good consolidation, with no pores detected—see also results of density measurements. It has been noted that the addition of DE ceramic particles results in a reduction of the size of grains in the metallic matrix, which is in good agreement with^7^. SEM examinations revealed that DE-rich particles are characterized by a highly developed surface and irregular shapes. This indicates relatively poor wetting of DE by the metallic matrix. The DE particles are located in between the grains of the Ti6Al4V—Fig. 2B, filling out the space available during sintering. Taking into account size of individual frustules and size of DE particles in the composites with linear dimensions, c.a. 30 µm, one can conclude that the particles are agglomerates of the frustules.

**Figure 3:**
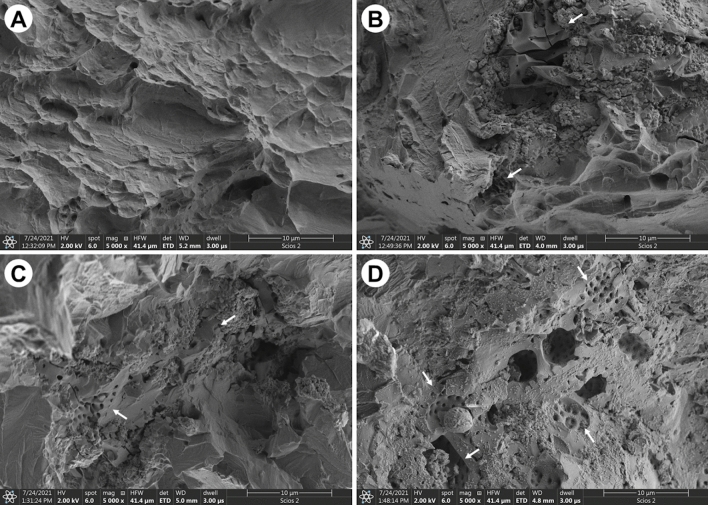
SEM images of fracture surfaces in the composite specimens: (**a**) Ti6Al4V alloy, (**b**) Ti6Al4V/1%DE, (**c**) Ti6Al4V/5% DE, (**d**) Ti6Al4V/10% DE; *white arrows—in-prints of diatom frustules in the metallic matrix.*

The SEM image of the fracture surface of the Ti6Al4V alloy (Fig. 3A) shows the presence of many ductile dimples and ductile tearing ridges, which suggest a ductile mode of failure. Fracture surface topography of the composites indicates that DE addition reduces ductility.

Good bonding between matrix and filler can inferred from SEM images, e.g., Fig. 3, Figure S2—Figure S4. In-prints of frustules are clearly seen and intact frustules can be found in composite samples (white arrows, Fig. 3; see also Figure S2—Figure S4 in Supplementary Information). The SEM images revealed that diatom frustules are empty inside and can be treated as a “caged pores”, see Figs. 2, 3.

#### X-ray diffraction analysis

**Figure 4:**
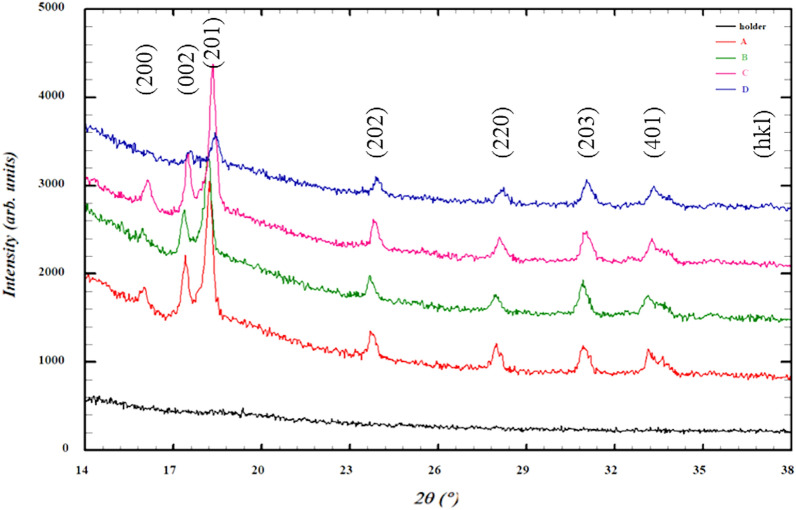
X-ray diffractograms of the sintered Ti6Al4V alloy composites with and without biogenic filler. Series: A—0%, B—1%, C—5%, D—10%.

Obtained XRD diagrams are shown in Fig. 4. It can be noted that no peak is observed of a V compound. This indicates that V is in solid solution. Oxygen has been found in TiAl2O5 (ISCD no. 98-015-4474) that exhibits hexagonal type structure (P 63/mmc no. 194). Addition of DE results in the shift to the right of the Bragg peaks. This might be an indication of residual strains generated in the composite matrix. Such strains are expected due to the mismatch in the coefficients of thermal expansion of Ti–rich matrix and DE particles. It can be noted that with the increasing volume fraction of DE, some diffraction peaks of Ti are reduced significantly because of the rising residual stresses and decreased fraction of a crystalline phase (Ti–rich) phase.

#### Contact angle analyses

The results of the investigations of surface wettability via measurements of the contact angle for sintered Ti6Al4V alloys with and without diatomaceous earth as a reinforcement are presented in Table 1 and Figure S5 (see Supplementary Information). The addition of the DE causes insignificant changes in the contact angle towards reducing hydrophilicity (from 54.56 for the pure alloy to 57.66 for 10% of reinforcement).

**Table 1:** The average values with a standard deviation of the contact angle of the sintered Ti6Al4V alloy composites with and without biogenic filler. Series: A—0%, B—1%, C—5%, D—10%.

Series	Wetting angle [º]
A	54.56 ± 1.29
B	55.54 ± 2.35
C	57.06 ± 1.58
D	57.66 ± 2.28

#### Thermo-mechanical properties

**Figure 5:**
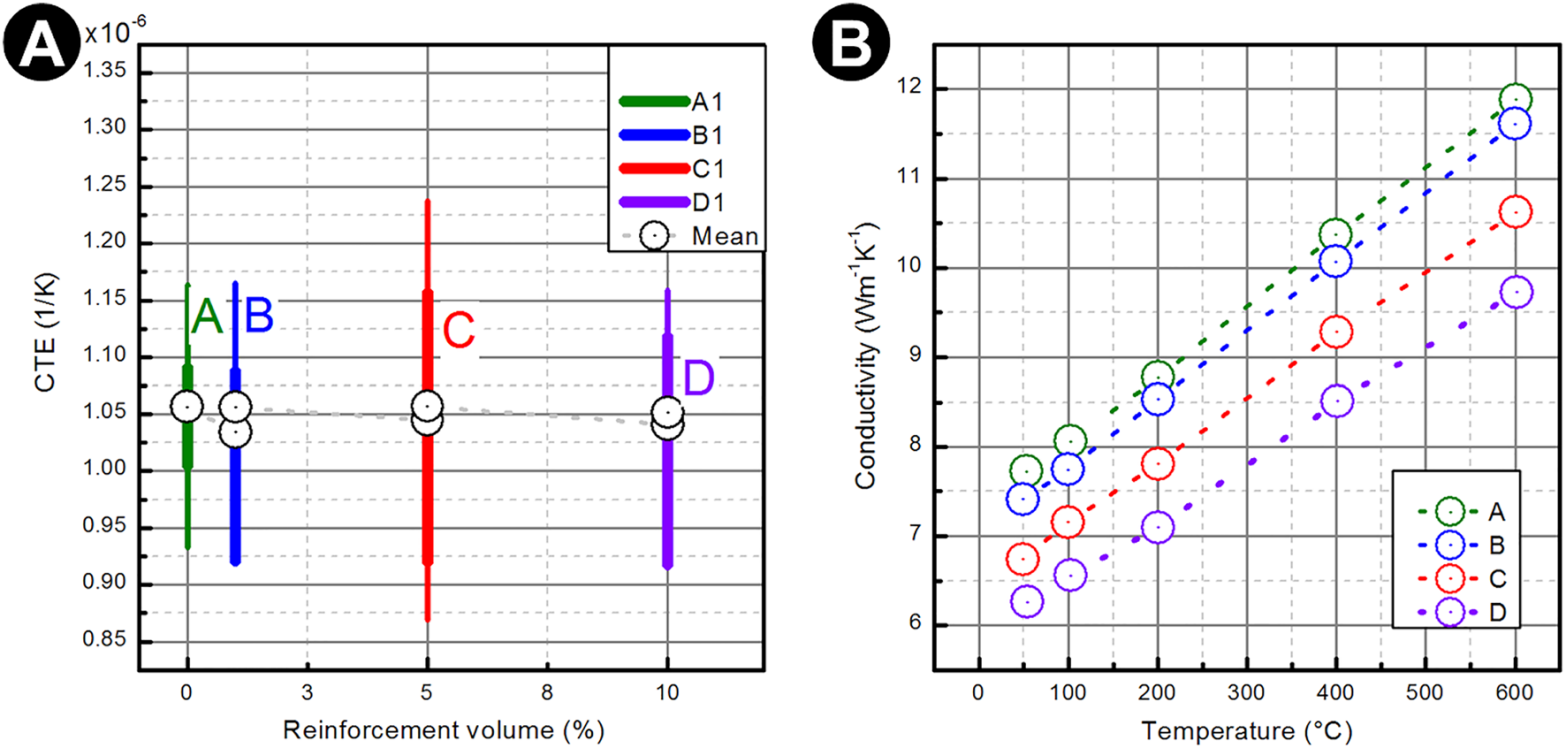
(**A**) The coefficient of the thermal expansion of the sintered Ti5Al4V alloy composites with and without biogenic filler. (**B**) The thermal conductivity as a function of temperature for sintered Ti6Al4V alloy composite with and without biogenic filler. Series: A—0%, B—1%, C—5%, D—10%.

The coefficient of thermal expansion (CTE) and thermal conductivity ($\lambda$) were obtained for the manufactured sample. Results are presented in Fig. 5A.

The importance of the coefficient of thermal expansion (CTE) of investigated samples is strictly related to the thermal properties of the material that affect the generation of tensile residual stresses. The coefficient of thermal expansion of ceramic reinforcement, regardless of its form, is smaller than that of most metallic matrices. Because of that lower coefficient, the thermal stresses, when the composite is subjected to temperature change, will be generated in both components—matrix and reinforcement. The predicted thermal properties are hard to realize due to the structure of the composites, interface as well as plastic deformation of the matrix due to internal thermal stresses.

The results show that the addition of the ceramic filler in the form of the diatomaceous earth does not cause major changes in mean apparent values of CTE for the composite specimens. On the other hand, CTE values for manufactured MMCs are characterized by a significantly higher dispersion of the measured values in comparison to a pure Ti6Al4V alloy.

The graphic depiction of the thermal conductivity (Fig. 5B) clearly shows an increase in conductivity with the temperature. The addition of the ceramic reinforcement results in lowering the conductivity, which is in good agreement with theory.

#### Mechanical properties

**Figure 6:**
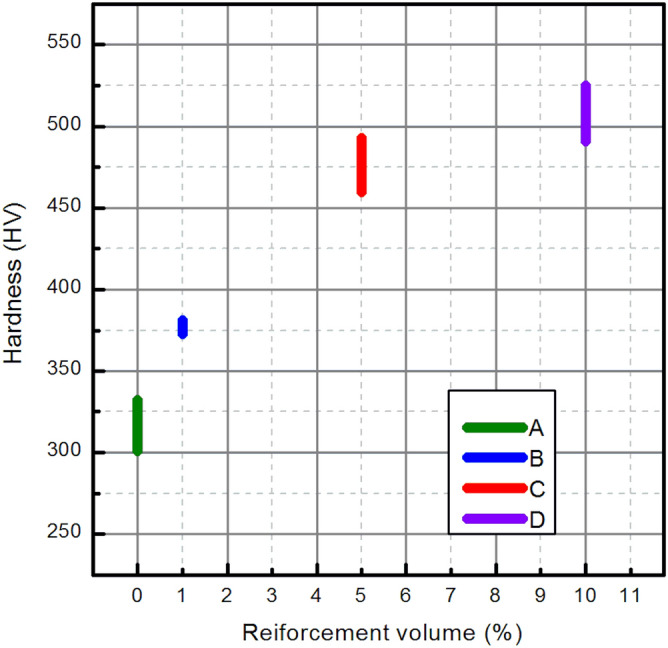
Hardness of Ti6Al4V based composites plotted against the content of the ceramic reinforcement.

The effect of the various volume fractions of the ceramic filler on micro-hardness is presented in Fig. 6 and in Table 2. It is observed that reinforcement of Ti6Al4V with diatomaceous earth increases the microhardness, from 314.96 HV, up to 378.37 HV and 512.29 HV, for 5 and 10% DE, respectively. It should be noted that an addition of 1% reinforcement results in significantly increase (20%) of hardness.

**Table 2:** Results of the hardness measurements via Vickers method. Series: A—0%, B—1%, C—5%, D—10%.

Series	HV5
A	314.96 ± 13.07
B	378.37 ± 3.33
C	476.79 ± 13.35
D	512.29 ± 13.80

Hayat et al*.* (2019) reported that the incorporation of hard ceramic particles in ductile titanium matrix significantly enhance its hardness^10^. Based on the graphical representation of the results obtained herein, departure from the rule of mixing is observed in the current case, indicating overlapping of strengthening and softening effects of DE reinforcement.

**Figure 7:**
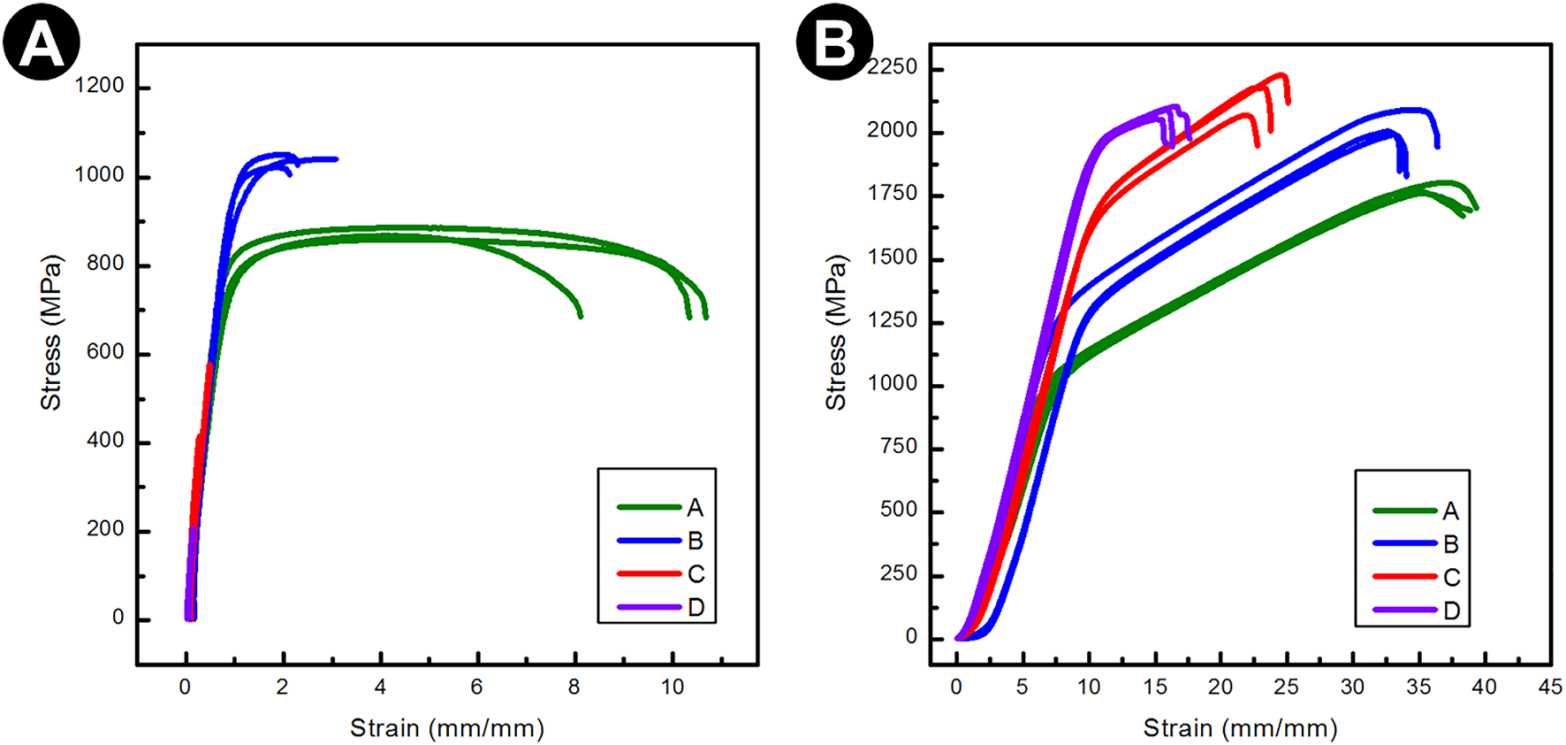
Stress–strain curve of manufactured composites Ti6Al4V/DE from the (**A**) tensile test, (**B**) static compression test. Series: A—0%, B—1%, C—5%, D—10% content of the ceramic reinforcement.

Results of the tensile test are presented in Table 3 and Fig. 7A. Compared to pure Ti6Al4V, TMC with 1% diatomaceous earth demonstrated higher strength parameters (both yield strength R_0.2t_ and tensile strength R_mt_). On the other hand, for 5 and 10 vol% of reinforcement, a decrease in tensile strength was observed. In fact specimens, with these volume fractions of DE, (5 and 10%) fractured below reaching a yield strength, exhibiting properties typical of ceramics. This is a clear indication that above 5% of volume fraction of DE, DE particles act as stress concentrators causing fracture prior to reaching yield point.

**Table 3:** Results of the static tensile test. Series: A—0%, B—1%, C—5%, D—10% content of the ceramic reinforcement.

Series	R_0.2t_ [MPa]	R_mt_ [MPa]	A_t_ [%]
A	767 ± 20	871 ± 11	9.00 ± 1.20
B	968 ± 35	1038 ± 12	1.34 ± 0.13
C	–	522 ± 75	0.08 ± 0.02
D	–	187 ± 24	0.03 ± 0.01

Fracture surface SEM images are presented in Supplementary Information (see Figure S6). The fracture surface of pure Ti6Al4V alloy shows various sizes of dimples. The addition of the reinforcement in the form of diatomaceous earth results in a mixture of brittle and ductile areas. The planar facets reveal in-prints caused by detachment of frustules present in DE.

Figure 7B shows the compressive stress–strain curves of the spark plasma sintered samples, whereas the values are presented in Table 4. The stress–strain curves show typical elastic–plastic deformation. An increase in the amount of the filler (1% and 5%) results in an increase in compression strength. The highest compression stress of 2159 MPa was obtained for the specimens with 5% of filler.

**Table 4:** Results of the static compression test. Series: A—0%, B—1%, C—5%, D—10% content of the ceramic reinforcement.

Series	R_2c_ [MPa]	R_mc_ [MPa]	A_c_ [%]
A	1111 ± 9	1778 ± 18	38.9 ± 0.40
B	1384 ± 7	2030 ± 44	34.6 ± 1.30
C	1801 ± 27	2159 ± 66	23.9 ± 0.90
D	2009 ± 7	2083 ± 21	16.6 ± 0.80

The result of the tests of mechanical properties of specimens can be discussed in terms: (a) effect of DE addition on the properties of Ti–rich matrix and (b) reaction of the composite structures to the applied load in tensile/compression. With regard to the effect of DE addition on the metallic matrix, it can be noted that composite samples are characterized by a smaller grain size of Ti crystals, which brings about a higher value of the yield flow of the composite matrix. The mechanism responsible for the size of grains in the Ti–rich matrix is very likely blocking of grain growth during sintering by DE particles. In fact, microstructures shown in Fig. 2 clearly show that DE particles are located in boundaries of Ti crystals. In addition to grain size refinement on the strengthening of Ti-matrix, one should also take into account the effect of geometrically-necessary dislocations needed to accommodate differences in the thermal contraction of the Ti-matrix and DE particles upon cooling from sintering temperature. In summary, presence of DE strengthens metal matrix in the composites of interest. On the other hand, DE particles have much lower mechanical strength than the Ti matrix and their presence reduces effective load bearing cross-sections of the specimens and thus brings about a reduction of composite strength. DE particles act also as stress concentrators, promoting brittle fracture of specimens in tensile tests. Thus, depending on the mode of applied load (tensile, compression, Vickers hardness) mechanical properties of the composite are determined by interplay between strengthening and weakening impact of DE particles. Strengthening effects dominate for small volume fractions and for compression mode. Recognition of these dichotomies on the impact of DE particles allows for selecting their appropriate volume fractions to given applications of the composites in question.

## Conclusions

The novel composites Ti6Al4V/diatomaceous earth have been fabricated using the spark plasma sintering (SPS) method. SEM images of these composites revealed that this technological route preserved the diatoms. The matrix does not penetrate the reinforcement, and good bonding between matrix and biogenic filler has been obtained. The increase of DE content within composites results in a decrease of the hydrophilicity—towards hydrophobicity.

The XRD measurements allowed us to identify the TiAl2O5 phase. No silicide phase was found. XRD spectra also revealed residual stresses generated by particles of DE.

Compared with samples sintered without filler, with the increase of DE content, the compressive yield strength increased while the plasticity gradually decreased. Especially for Ti6Al4V-5 vol% DE composite, the compressive yield strength is 1801 MPa, which is ca. 62% higher than that of pure Ti6Al4V (1111 MPa). Additionally, it maintains good compressive plasticity (34.6%). In the case of the tensile test, the highest values have been obtained for Ti6Al4V-1 vol% DE composite, for which the tensile yield strength was 968 MPa, which is ca. 26% higher than that of pure titanium (767 MPa) and maintain acceptable tensile strain.

In the case of the mean values of the coefficient of thermal expansion (CTE), no significant differences for pure Ti6Al4V alloy and with the addition of ceramic filler (DE) have been noticed. The thermal conductivity has been lowered with the addition of diatomaceous earth.

Comparison of our results with the values presented in the literature clearly show that the samples with 1 vol% and 5 vol% DE demonstrated better tensile strength than elements sintered by the BE method. At the same time compression strength exceeds 2000 MPa. This is remarkably high value making the composites fabricated here a promising candidate for manufacturing of devices in which applied loads primarily generate compressive stresses.

The results show that biogenic filler in the form of diatom frustules can be used as an attractive reinforcement for future applications in development of high-performance TMCs, e.g., for aerospace, automotive, and sporting goods.

## Experimental

### Materials

Ti6Al4V (UNS R56400/3.7165), Titanium Grade 5, spherical powder (Wolften, Wroclaw, Poland) with a particle size: 0–53 µm (density 2.53 g/cm^3^) and 53–105 µm (density 2.56 g/cm^3^) was used. Diatomaceous earth (DE, Diatomite, Perma-Guard) consisting of shells of unicellular microscopic organisms (*Aulacoseira* sp.) from the extremely clean freshwater deposits was used as a filler.

### Manufacturing of composites

Mixtures of Ti6Al4V and diatomaceous earth (DE) were used in the experimental part. The SPS process was conducted under vacuum at the uniaxial compressive pressure of 1.2 MPa. The powder mixtures were placed in cylindrical graphite dies with an inner diameter of 25 mm and pressed between two graphite punches. The mixtures were heated up to 1000 °C with a heating rate of 50 °C × min^−1^ and maintained at final temperature for 5 min. The process has been conducted in inert gas—Argon at − 0.5 Atm pressure. The relative density of the samples, determined by the Archimedes method, was estimated at 100% of the theoretical value.

### Experimental techniques

#### Characterization of powders and consolidated samples

Characterization of Ti6Al4V powder and diatomaceous earth DIATOMIT (Perma-Guard, USA) was carried out using an ultra-high-resolution analytical dual-beam FIB-SEM tool (Scios2 DualBeam, Thermo Fisher Scientific, Waltham, MA, USA). Powder samples were coated with Au (5 nm layer) using a high-vacuum sputter coater. Elemental analyses of Ti6Al4V powders and diatomaceous earth were carried out using Energy Dispersive X-ray Spectroscopy (EDS). Elemental maps were collected under an acceleration voltage of 30 kV, elemental range of 10 keV.

The grain size distribution of the Ti6Al4V powder was measured by a Laser Particle Size Analyzer (Fritsch, Idar-Obserstein, Germany) in water suspension. The distribution of the size of diatom frustules was measured using Air Jet Sieving Machine AS200 jet (Retsch, Germany) during the fractionation process.

The experimental density (bulk density) of the composites was obtained by the Archimedes method. The theoretical density was calculated using the rule of mixture. Bulk density was calculated by using Eq. (1):

1 $${\rho }_{B}= \frac{{m}_{d}}{{m}_{sat}-{m}_{sus}}\cdot {\rho }_{{H}_{2}O}$$

where *ρ*_*B*_—the bulk density, m_sat_—saturated mas, m_d_—dry mass, m_sus_ – suspended immersion mass.

X-ray diffraction (XRD) measurements at room temperature were performed using an Empyrean Panalytical powder diffractometer equipped with Mo X-ray tube (*K*_*α*_ radiation, λ = 0.7093187 Å, 40 kV, 40 mA) and PixCel1D strip detector. The scattered intensity was recorded in the Bragg–Brentano geometry in a range of 2θ from 14° to 38° in steps of 0.026261°. The pattern acquisitions have been conducted on solid samples (4 × 4 mm) on a plexiglass holder. The small area (4 × 4 mm) of solid samples required a narrow fixed slit. The change of the aperture led to the much smaller response from the background (plexiglass cover) in the diffractogram. The phase analysis was carried out based on the ISCD database using the HighScore program^47^.

Contact angle measurements were performed by the sessile drop technique at room temperature and atmospheric pressure, with a Osilla Contact Angle Goniometer (Osilla, Sheffield, UK). Ten independent measurements were performed for each sample, each with a 2 µl water drop. In order to examine the macroscopic characteristic and to eliminate the effect of topography, the measurements of contact angle have been conducted on the polished cross-sections. The obtained results were averaged to reduce the impact of surface nonuniformity.

The hardness of the composite samples was tested by the Vickers method using a Hardness Tester DuraScan 20 (Struers) with the load HV5 (ca. 49.03 N) according to the PN-EN ISO 6507-1.

The coefficient of thermal expansion, CTE, was measured using the standard four-probe method in a vacuum. The thermal conductivity, $\lambda$, was calculated according to the formula $\lambda = \alpha \cdot {C}_{p}\cdot \rho$, where C_p_ is the theoretical heat capacity based on the measurements via laser flash method (LFA, Netzsch, 457 MicroFlash) using a sample with a diameter of 10 mm and height of 1 mm. All measurements were performed over the temperature range of 323 K to 723 K.

The static tensile tests were carried out using the Small-Specimen Tensile Test (SSTT) technique^48–50^. Samples with the dimensions given in Supplementary Information (see Figure S1) were tested with a Zwick/Roell Z005 (ZwickRoell GmbH & Co. KG, Germany) universal electromechanical testing machine equipped with a load cell having a load capacity of ± 1 kN. The tests were controlled by a crosshead displacement of the testing machine of 0.005 mm/s. The initial strain rate was 1 × 10^–1^ 1/s.

Because of the small size of the testing specimens, a non-contact optical method based on image correlation was applied (DIC-Digital Images Correlation) for strain calculations. Local deformations, and the fields of deformations over the entire area of a tested specimen, were analyzed using VIC 2d commercial software provided by Correlated Solutions Inc. The values of the engineering stress–strain curves were calculated by post-processing DIC analysis. For the static compression tests, cube specimens with a characteristic dimension of 3 mm were used. The test was controlled by constant displacement in time (0,003 mm/s), and the strain rate was finally the same as in tensile 1 × 10^–1^ 1/s. For the compression tests, non-standard yield strength at 2% of the plastic strain was calculated.

## Supplementary Information

Supplementary Information.

## Data Availability

All data generated or analysed during this study are included in this published article (and its Supplementary Information files).
