# Supplementary material for: Titanium matrix composites reinforced with biogenic filler
Source: Sci Rep. 2022 May 24;12:8700. doi: 10.1038/s41598-022-12855-5 (PMC9130303; doi:10.1038/s41598-022-12855-5)
Supplement: Supplementary file 1 — Supplementary Information. [file 41598_2022_12855_MOESM1_ESM.docx]

**Supplementary Information**

**Titanium matrix composites reinforced with biogenic filler**

Izabela Zglobicka, Rafal Zybala, Kamil Kaszyca, Rafal Molak, Monika Wieczorek, Katarzyna Recko, Barbara Fiedoruk, Krzysztof J. Kurzydlowski

**Figures**


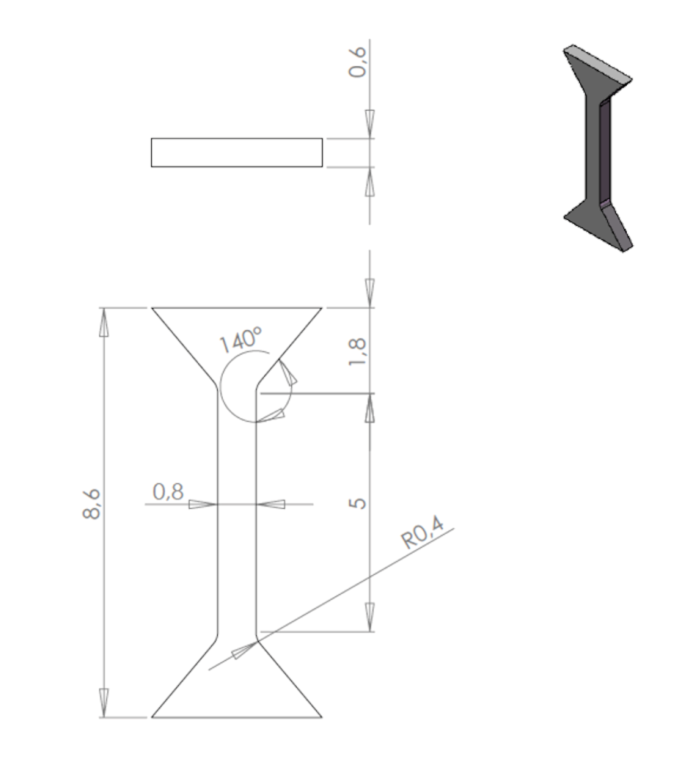


Figure S1: Shape and dimensions of the specimen used for the static tensile tests


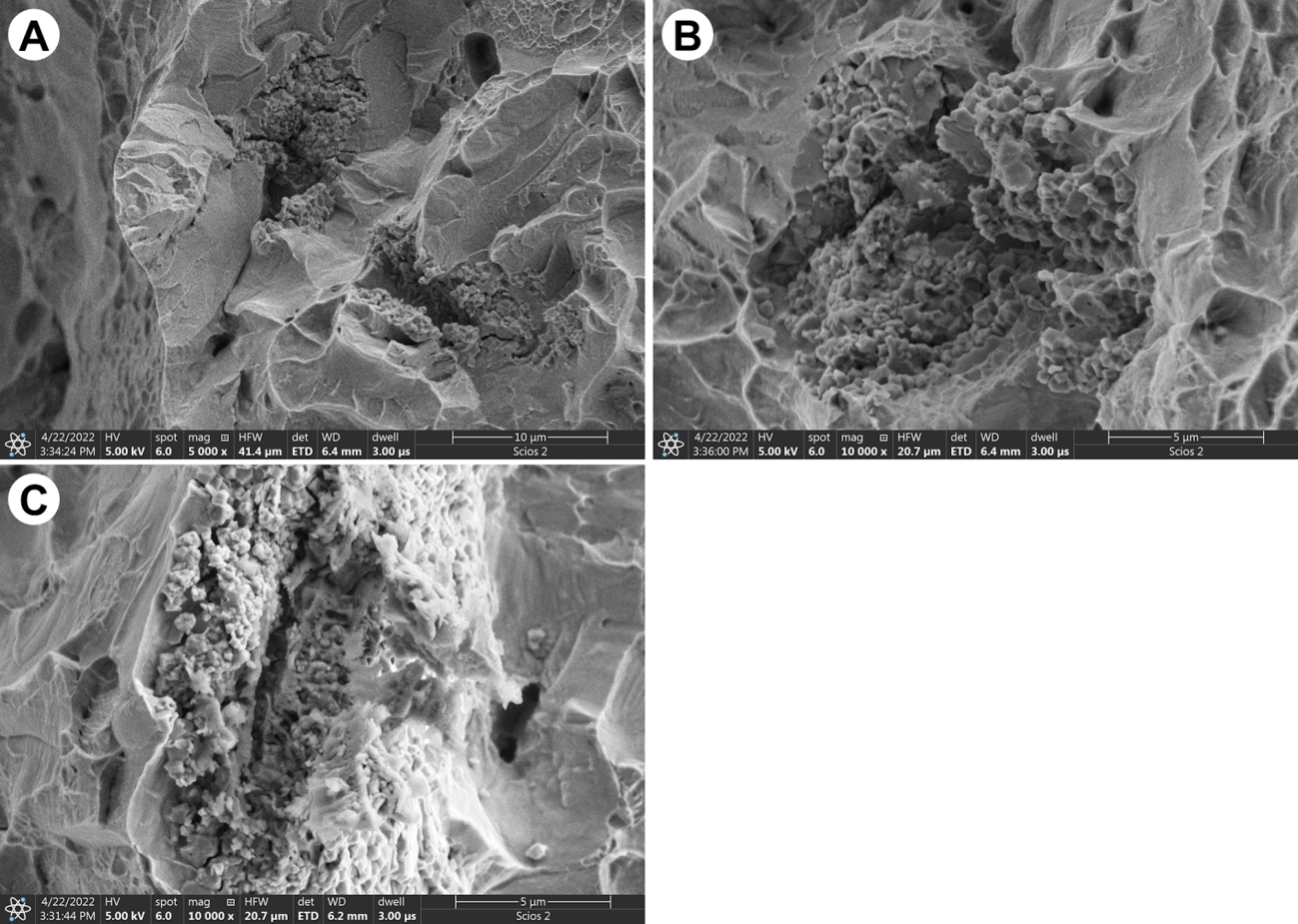


Figure S2: SEM images of the fracture surfaces of composite Ti6Al4V/1 vol% DE


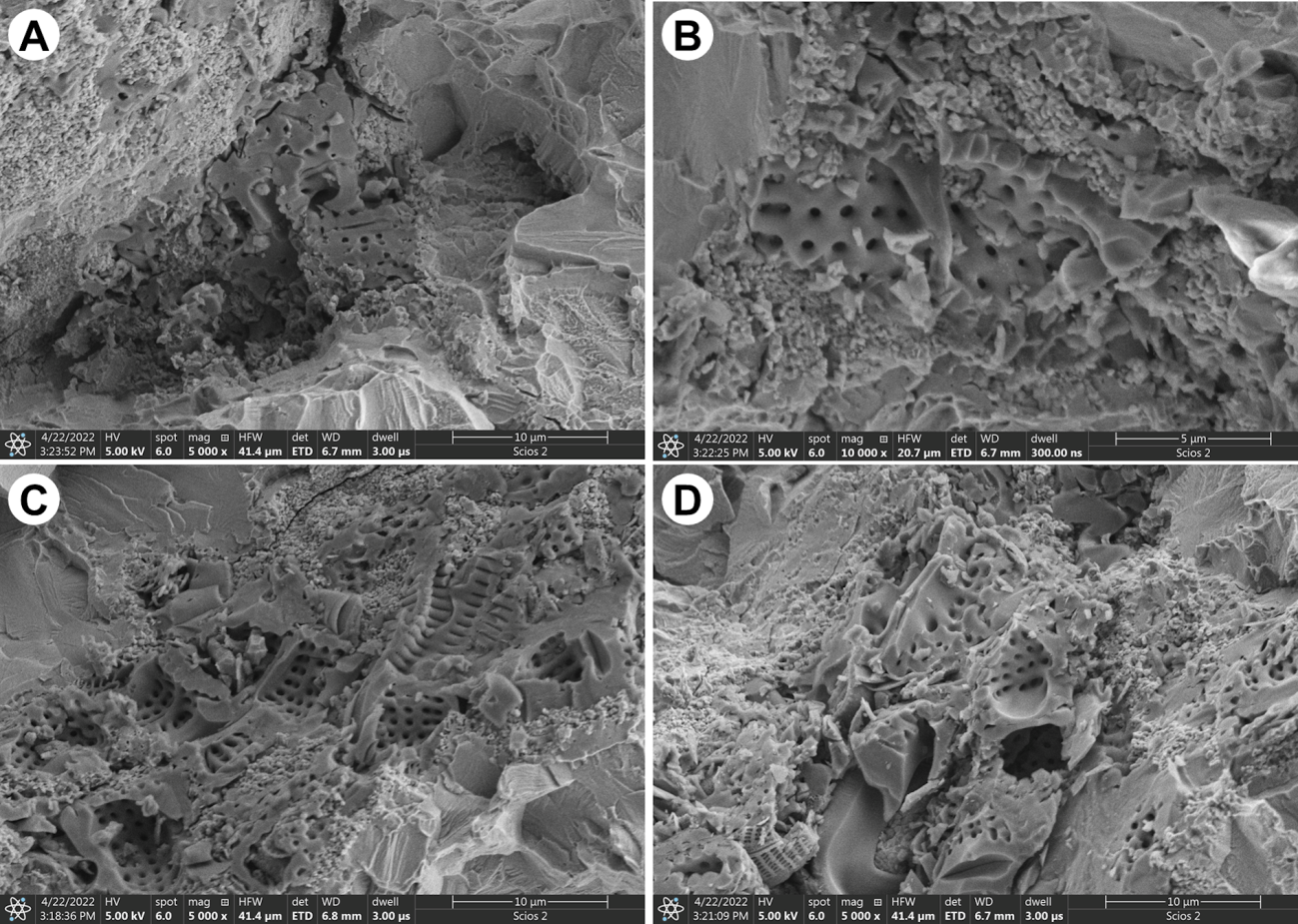


Figure S3: SEM images of the fracture surfaces of composite Ti6Al4V/5 vol% DE


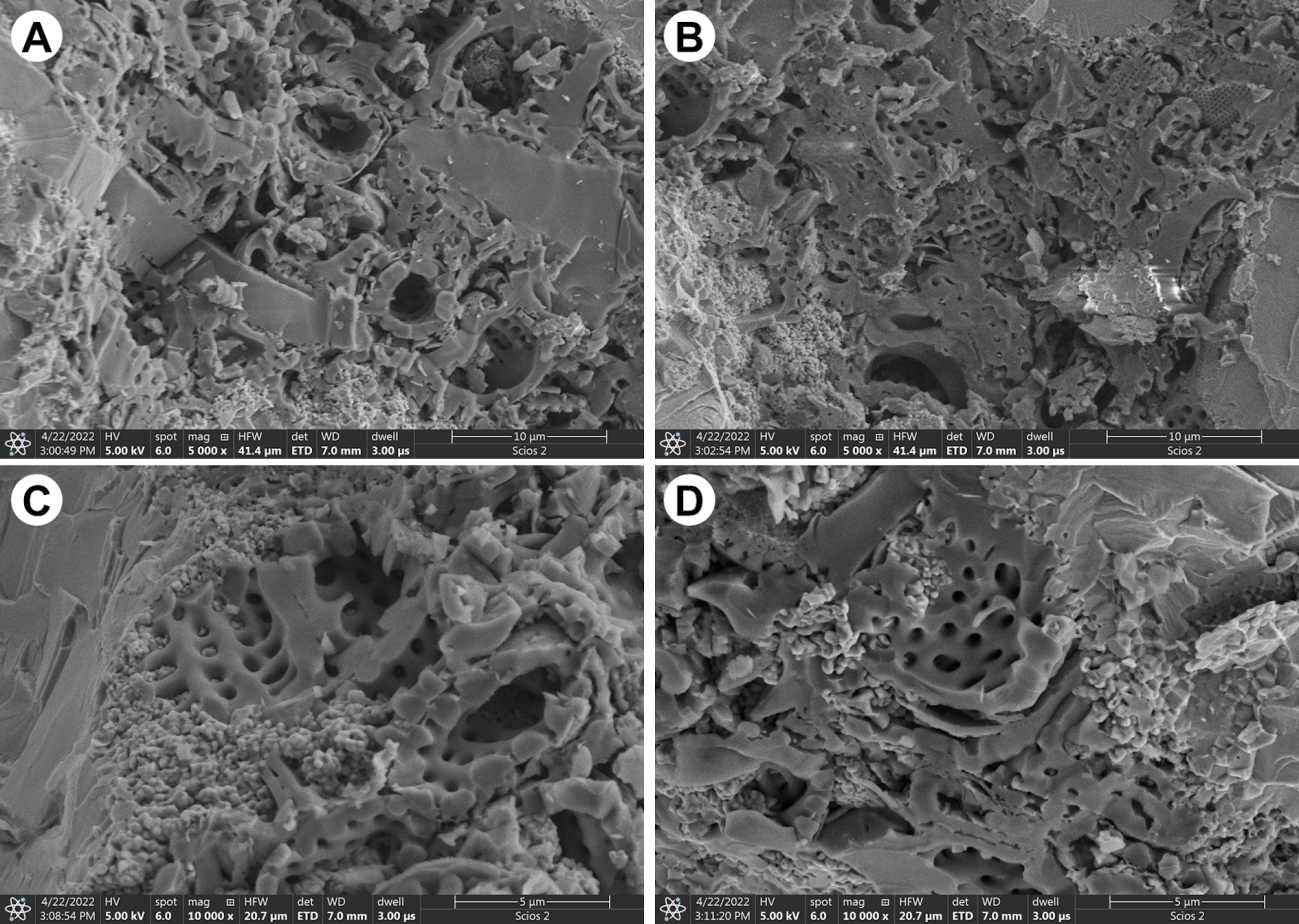


Figure S4: SEM images of the fracture surfaces of composite Ti6Al4V/10 vol% DE





Figure S5: Contact angle variations of the sintered Ti6Al4V alloy composites with and without biogenic filler. Series: A – 0%, B – 1%, C – 5%, D – 10%


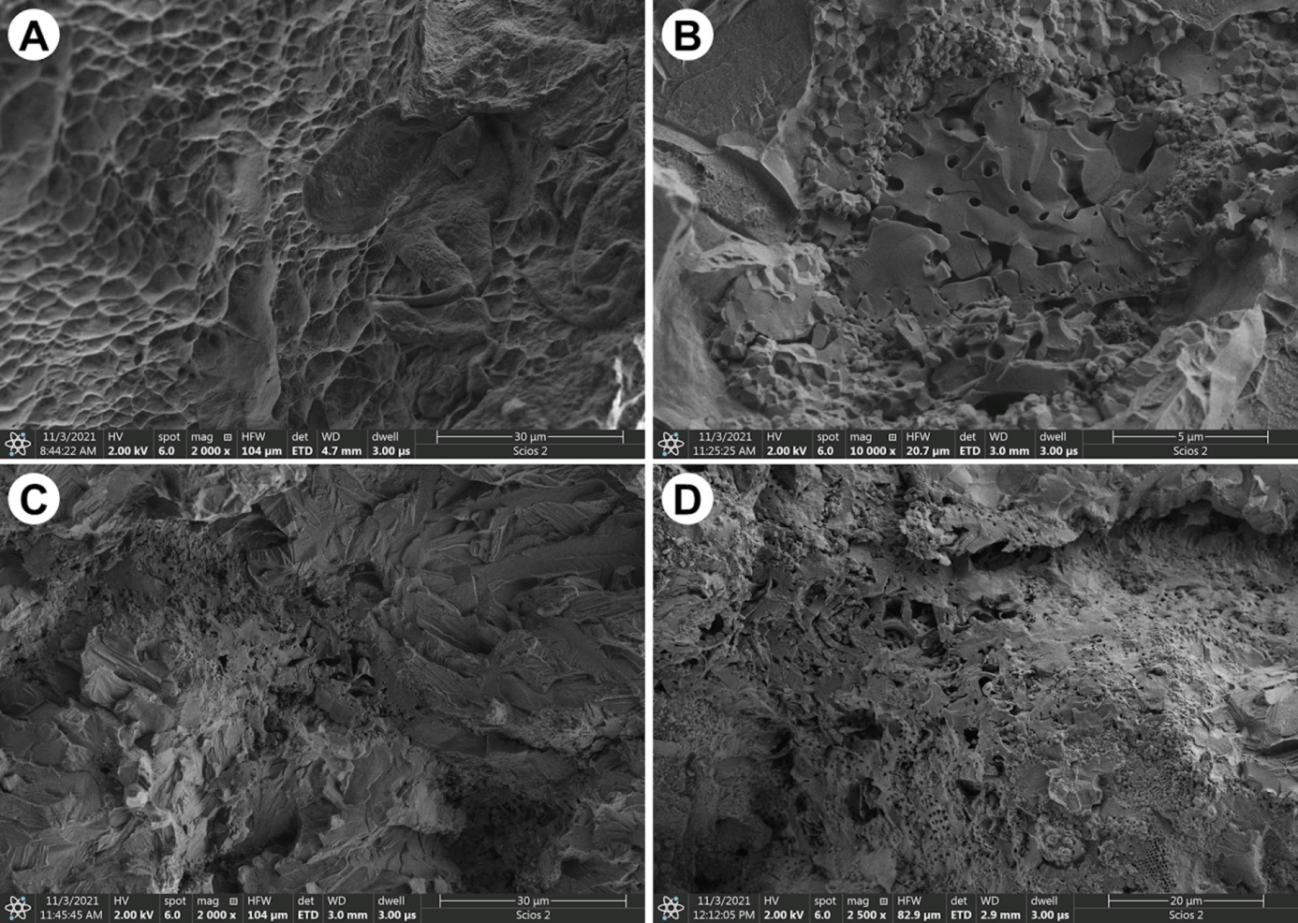


Figure S6: SEM images of the fracture surfaces of composites after tensile test. Series: A – 0%, B – 1%, C – 5%, D – 10%

**Tables**

Table S1: Theoretical and experimental density of the manufactured samples

| Ti6Al4V [vol%] | DE [vol%] | ρ_th_ [g/cm^3^] | ρ_exp_ [g/cm^3^] |
| --- | --- | --- | --- |
| 100 | 0 | 4.430 | 4.398 |
| 99 | 1 | 4.409 | 4.398 |
| 95 | 5 | 4.324 | 4.372 |
| 90 | 10 | 4.217 | 4.292 |
